# Association of plasma growth differentiation factor-15 with the frailty phenotype – Evidence from the COGFRAIL study

**DOI:** 10.1016/j.tjfa.2026.100194

**Published:** 2026-09-04

**Authors:** Federico Bellelli, Juan Luis Sanchez Sanchez, Davide Angioni, Alexandre Lucas, Yves Rolland, Marco Proietti, Bruno Vellas, Philipe De Souto Barreto, Sandrine Sourdet

**Affiliations:** a IHU HealthAge, Toulouse, France; b Institut du Vieillissement, Gérontopôle de Toulouse, Centre Hospitalo-Universitaire de Toulouse, Toulouse, France; c CERPOP, Inserm 1295, Toulouse University, INSERM, UPS, Toulouse, France; d Institut National de la Santé et de la Recherche Médicale (INSERM), UMR 1297, Institute of Metabolic and Cardiovascular Diseases, Toulouse, France; e Department of Geriatrics, Hospital and University of Toulouse, Toulouse, France; f Department of Clinical Sciences and Community Health, University of Milan, Milan, Italy; g Division of Cardiogeriatric Subacute Care, IRCCS Istituti Clinici Scientifici Maugeri, Milan, Italy; h Gérontopôle, Department of Geriatrics, Toulouse University Hospital, La Cité de la Santé, Hôpital La Grave, Toulouse, France

**Keywords:** Frailty, Sarcopenia, Weight loss, Appetite, Growth differentiation factor 15

## Abstract

**Background:**

Growth differentiation factor-15 (GDF-15) has been consistently associated with physical frailty. However, it remains unclear whether GDF-15 further discriminates adverse outcomes among older adults who are already physically and cognitively impaired.

**Objectives:**

To evaluate whether GDF-15 is associated with measures of physical frailty - incident weight loss, decline in muscle strength and gait speed, fatigue, and sedentary behaviours - over a 2-year follow-up.

**Design:**

Monocentric prospective study

**Setting:**

Frailty and Memory Clinics, Gérontopôle Toulouse University-Hospital.

**Participants:**

206 pre-frail and frail older adults with cognitive impairment from the COGFRAIL study with available plasma GDF-15 concentrations (median age 82.0). Secondary analyses were performed in a subgroup with repeated GDF-15 measurements during follow-up (n = 129).

**Measurements:**

GDF-15 concentrations were analysed as a continuous variable and by tertiles. Exploratory analyses used two thresholds: 1500pg/mL (eligibility cut-off for GDF-15 inhibitor treatment in cancer-cachexia) and 2443pg/mL (upper tertile of the 6-month distribution).

**Results:**

Higher GDF-15 levels were associated with a steeper decline in handgrip strength over time (*p* = 0.047), with no significant associations for weight loss, gait speed, sedentary behaviour, or fatigue. By tertiles, participants in the highest tertile had greater handgrip strength decline compared to those in the lowest (*p* = 0.018). Participants in the intermediate group had a higher risk of worsening frailty (*p* = 0.013) and incident weight loss (SHR 2.51; 95% CI: 1.04–6.04; *p* = 0.040) compared with the lowest tertile. Individuals with persistently high or increasing GDF-15 concentrations (≥2443pg/mL) experienced greater decline in handgrip strength than those with persistently low levels (*p* < 0.001).

**Conclusions:**

Higher plasma GDF-15 concentrations consistently predicted a steeper decline in muscle strength over time. Associations with incident weight loss and worsening frailty were also observed, although they appeared weaker at higher concentrations, possibly because of greater competing mortality in participants with very high GDF-15 levels. Further research should clarify the mechanisms linking GDF-15 to physical decline and weight loss, and whether targeting this pathway could help slow frailty progression

## 1 Introduction

Physical frailty is a multi-dimensional syndrome characterised by decreased physiological reserve and increased vulnerability to adverse outcomes such as disability and mortality [1]. The underlying factors of frailty are interconnected and perpetuate a vicious cycle in which components like undernutrition and sarcopenia exacerbate each other, ultimately resulting in the clinical manifestation of frailty [2]. Fried et al. operationalised this cycle into a clinically recognisable phenotype defined by the presence of at least three of the following criteria: weakness, slowness, sedentary behaviours, fatigue, and unintentional weight loss [2]. Although frailty is a robust predictor of adverse outcomes, individuals classified as frail or pre-frail may still follow heterogeneous trajectories [3]. In this context, there is a need to identify biomarkers that can further refine risk stratification, even within populations already pre-frail or frail. Such biomarkers may help uncover pathways for therapeutic strategies aimed at disrupting - or even reversing - the cycle of frailty.

Growth differentiation factor 15 (GDF-15) is a stress-responsive protein belonging to the transforming growth factor-beta (TGF-β) superfamily. GDF-15 expression markedly increases in response to cellular stress and has been implicated in a wide spectrum of clinical conditions, including metabolic disorders, chronic inflammation, mitochondrial dysfunction and malignancies [4]. In cancer patients, elevated GDF-15 levels have been associated with weight loss, reduced muscle mass, and decreased strength [5,6] - pivotal features of physical frailty. In 2024, a phase II clinical trial showed that inhibition of GDF-15 with a humanised monoclonal antibody resulted in significant weight gain, improved appetite, and increased physical activity (PA) among individuals with cancer cachexia [7]. Although derived from a distinct clinical population, these findings suggest that targeting GDF-15 may have the potential to modulate key components of the frailty cycle. Over the past decade, multiple cross-sectional studies have consistently reported associations between higher circulating GDF-15 and physical frailty [[8], [9], [10], [11], [12]]. However, longitudinal evidence remains limited. A 2026 study reported that GDF-15 concentrations (>1312.0 pg/mL) predicted an increased risk of incident frailty and weight loss over a 4-year follow-up in a population of relatively healthy older adults [13]. Nevertheless, it is still unclear whether higher GDF-15 levels can further discriminate the risk of adverse outcomes among older individuals who are already cognitively and physically impaired.

This study aimed to evaluate whether plasma GDF-15 levels are longitudinally associated with measures of physical frailty over a two-year follow-up in a cohort of pre-frail and frail older adults with objective cognitive impairment. Because GDF-15 may transiently increase in response to acute stress, secondary analyses were restricted to participants with measurements at both 6 and 24 months, as persistent elevation across both time points was considered more likely to reflect a chronic increase.

## 2 Methods

### 2.1 Study design and participants

This is a secondary analysis of longitudinal data from the Cognitive Function and Amyloid Marker in Frail Older Adults (COGFRAIL) cohort (registration: NCT03129269). In brief, COGFRAIL is a prospective, single-center observational cohort study designed to estimate amyloid positivity prevalence in older adults with early cognitive impairment. Participants were community-dwelling individuals aged 70 years or older presenting with mild cognitive impairment or mild dementia and exhibiting at least one frailty criteria: unintentional weight loss, fatigue, weakness, slowness, or low physical activity. Individuals meeting three or more of these criteria were categorized as frail; those with one or two criteria were considered pre-frail [2]. Exclusion criteria included severe somatic or psychiatric conditions or required assistance with more than two Activities of Daily Living (ADL). Full methodology of the COGFRAIL study has been described elsewhere [14]. Ethical approval was granted by the institutional review board (Registration Number: RC31/16/8753), and all participants gave written informed consent in accordance with the Declaration of Helsinki.

Recruitment occurred between January 2017 and February 2020 during routine outpatients’ visits at the Frailty Clinics or Memory Clinics of the Gérontopôle, Toulouse University Hospital, or as part of community-based clinical evaluations. Participants were referred by general practitioners, specialists, or geriatricians for standard cognitive or frailty assessments. Follow-up procedures aligned with the standard clinical care typically provided in these units.

The analytical baseline was defined as the first COGFRAIL visit, where the most complete clinical and frailty-related assessment was available. Because GDF-15 was not measured at baseline, the study sample was restricted to participants with available GDF-15 measures at the 6-month visit, yielding a final sample of 206 individuals. Therefore, baseline clinical characteristics refer to the first visit, while GDF-15 concentrations refer to the 6-month assessment. Among these participants, 129 also had a follow-up GDF-15 assessment at two years.

### 2.2 Plasma GDF-15 measurement

Fasting blood samples for plasma GDF-15 measurement were collected at the 6-month (n = 206) and 2-year (n = 129) follow-up visits and stored at -80 °C until analyses. Plasma GDF-15 concentrations were measured using the Ella automated immunoassay system (Bio-Techne, San Jose, CA, USA), which employs a microfluidic cartridge-based approach. Further methodological details are available elsewhere [15]. Each disposable SimplePlex cartridge contains analyte-specific glass nanoreactors per detection channel, allowing for triplicate measurements of GDF-15 per sample. Before analysis, plasma aliquots were thawed on ice and diluted at a 1:10 ratio with sample diluent (SD 13). Samples and both high and low concentration controls were loaded into the cartridges. The assay includes an integrated, lot-specific standard curve embedded within the cartridge, enabling automated quantification. All procedural steps were carried out by the Ella system, and final GDF-15 levels were reported in pg/mL using the system’s built-in analysis software.

In the absence of established cutoffs for abnormal plasma GDF-15 levels in this specific population, GDF-15 concentrations were analysed both as a continuous variable and by tertiles (based on the within-group distribution). In addition, exploratory analyses were conducted using the 1500 pg/mL threshold, previously identified as the eligibility threshold for GDF-15 inhibitor treatment in individuals with cancer cachexia [7], and the upper tertile of the 6-month distribution (2443 pg/mL).

### 2.3 Measures of physical frailty

The five criteria of physical frailty were evaluated as follows:

1) In accordance with previous literature [16], weight loss was defined as a ≥ 5% reduction in body weight over six months. Data on weight loss were collected at each 6 months visit (6–12–18–24 months).
2) Weakness was assessed using Handgrip strength of the dominant hand, measured in kilograms using a JAMAR hydraulic dynamometer (J. A. Preston Corporation, Clifton, NJ, USA). Participants performed three consecutive measurements while standing with the elbow fully extended, and the highest value was used for the analyses.
3) Slowness was evaluated by usual-pace gait speed with the 4-meter walk test (measured in meters per second).
4) Sedentary behaviour was measured using a self-reported 0–5 scale ranging from no activity (0) to vigorous activity several times per week (5). Because 97.6% of participants reported either very light activity (1) or light-intensity activity for 2–4 h per week (2), the scale was dichotomised by merging categories 0–1 into the sedentary group and categories ≥2 into the non-sedentary group.
5) Fatigue was measured using two self-reported questions on how often, during the past week, participants experienced the following: “Everything I did was an effort” and “I could not get going.” Each item was rated on a 4-point scale: 0 = rarely (<1 day), 1 = sometimes (1–2 days), 2 = often (3–4 days), and 3 = most of the time (≥5 days).

### 2.4 Statistical analysis

Descriptive statistics were presented as medians with interquartile ranges (IQRs) for continuous variables and frequencies with percentages for categorical variables. Baseline characteristics by GDF-15 status were compared using Student’s *t*-test, Mann–Whitney U test, or one-way ANOVA with Tukey’s post hoc test for continuous variables, and χ² test or Fisher’s exact test for categorical variables, as appropriate.

The number of frailty phenotype criteria and the five individual criteria (weight loss, handgrip strength, gait speed, sedentary behavior, and fatigue) were considered as primary outcomes. Fine–Gray subdistribution hazard models were used to assess the association between 6-month GDF-15 concentrations and incident weight loss, with follow-up time calculated from the initial COGFRAIL visit (M0). Death before weight loss was treated as a competing event, while participants without weight loss or death were censored at their last follow-up visit. Subsequently, a series of multivariable linear mixed-effects regression models with a random intercept at the individual level were used to investigate the association between GDF-15 concentrations and the number of frailty criteria, handgrip strength, fatigue and gait speed. To improve the distribution of residuals [17], gait speed was log-transformed. To account for sex differences in muscle strength, handgrip strength values were standardised into Z-scores using sex-specific means and standard deviations (SD) [18]. Finally, the association between GDF-15 concentrations and changes in sedentary behaviour (binary outcome) was examined using a logistic generalised linear mixed model with a random intercept at the individual level. All models were adjusted for age, sex, living situation (alone vs other), Baseline Mini-Mental State Examination (MMSE) score, and Charlson Comorbidity Index (CCI). The model for the number of frailty criteria was additionally adjusted for baseline frailty, whereas the weight loss model was additionally adjusted for baseline BMI. Mortality during follow-up was described across GDF-15 tertiles and compared using Fisher’s exact test.

Secondary analyses were restricted to participants with GDF-15 measurements at both 6 months and 2 years (n = 129) to capture persistent elevations better. In these analyses, GDF-15 was treated as a continuous variable (change over time) and categorised into four groups based on changes between the two measurements - “Persistently Low,” “Decreasing,” “Increasing,” and “Persistently High” - using the two exploratory thresholds (1500 pg/mL and 2443 pg/mL).

As sensitivity analyses, the association between GDF-15 concentrations and incident weight loss was also examined using standard Cox proportional hazards models, in which deaths were treated as censoring events. Estimates from the Fine–Gray and Cox models are presented side by side in Supplementary Table 1. In addition, given the tertile-based pattern observed for incident weight loss and the number of frailty criteria, potential non-linearity was explored for these two outcomes by comparing models using restricted cubic splines for continuous GDF-15 with the corresponding linear models using likelihood ratio tests.

Statistical analyses were performed with R statistical package Version 2023.12.1 + 402, with a significance level defined at *p* < 0.05.

### 2.5 Use of artificial intelligence

The authors used an artificial intelligence–based language model (ChatGPT, OpenAI) exclusively for language editing and grammatical refinement. All scientific content, data analysis, and interpretation were conceived, written, and validated by the authors.

## 3 Results

### 3.1 Characteristics of the population

The median age at baseline was 82.0 years (IQR: 78.3–86.0), and approximately two-thirds (n = 136, 66.0%) of participants were women. The median plasma GDF-15 concentration at 6 months was 1911.5 (IQR: 1419.25 to 2706.5) pg/mL. Baseline characteristics stratified by GDF-15 concentrations are presented in Table 1. Participants in the intermediate and highest tertiles were older and had higher CCI scores than those in the lowest tertile.

**Table 1:** Baseline characteristics of the population according to GDF-15 concentrations.

		**Tertiles**			
**Variables**	**Total population**	1st tertile	2nd tertile	3rd tertile	**p**
		GDF-15	GDF-15	GDF-15	
		(≤1570 pg/mL)	(1571–2443 pg/mL)	(>2443 pg/mL)	
N (%)	206	69 (33.5%)	68 (33%)	69 (33.5%)	/
Age, median (IQR)	82.0 (7.8)	80.0 (7.0)	82.0 (7.0)	83.0 (6.0)	**0.006**^a,b^
Female Sex, n(%)	136 (66.0%)	51 (73.9%)	41 (60.3%)	44 (63.8%)	0.216
Living alone, n(%)	76 (36.9%)	31 (44.9%)	23 (33.8%)	22 (31.9%)	0.231
Low Education	123 (60%)	41 (60.3%)	35 (51.5%)	47 (68.1%)	0.138
CCI†, median (IQR)	2.0 (3.0)	2.0 (2.0)	2.0 (3.0)	3.0 (3.0)	**<0.001**^a,b^
BMI (kg/m^2^), median (IQR)	26.0 (5.8)	25.9 (5.6)	25.1 (5.6)	26.5 (4.9)	0.384
MNA†, median (IQR)	25.0 (3.5)	25.5 (3.1)	25.5 (3.5)	25.0 (2.5)	0.114
Handgrip, median (IQR)	16.0 (9.8)	16.0 (9.0)	18 (10.2)	18.0 (10.0)	0.250
Frailty					
Pre-Frail, n(%)	111 (53.9%)	39 (56.5%)	42 (61.8%)	30 (43.5%)	0.086
Frail, n(%)	95 (46.1%)	30 (43.5%)	26 (38.2%)	39 (56.5%)	
Fried criteria					
Weight Loss, n(%)	13 (6.6%)	3 (4.5%)	6 (8.9%)	4 (6.5%)	0.594
Fatigue, n(%)	168 (81.9%)	55 (79.8%)	58 (86.6%)	55 (79.8%)	0.488
Weakness, n(%)	113 (54.8%)	40 (57.9%)	37 (54.4%)	36 (52.1%)	0.788
Sedentary behaviors, n(%)	153 (74.3%)	47 (68.1%)	48 (70.6%)	58 (84.1%)	0.070
Slow gait speed, n(%)	90 (44.1%)	27 (39.7%)	25 (37.3%)	38 (55.9%)	0.076
GDF-15 (pg/mL), median (IQR)	1911.5 (1287)	1323 (285)	1912 (362)	3345 **(**1309)	**<0.001**^a,b,c^
MMSE, median (IQR)	25.0 (5.0)	24.0 (5.0)	25.0 (4.0)	24.0 (4.0)	0.421
CDR-SB, median (IQR)	2.0 (2.0)	2.5 (2.5)	2.0 (1.5)	2.5 (2.5)	0.266

Legend: BMI, Body Mass Index; CCI, Charlson Comorbidity Index; CDR-SB, Clinical Dementia Rating Sum of Boxes; GDF-15, Growth Differentiation Factor 15; MMSE, Mini Mental State Examination; MNA, Mini Nutritional Assessment; PA, Physical Activity;.

‡Fisher’s Exact Test.

† Mann-Whitney U test.

Seventeen participants died during follow-up, with the highest mortality rate observed in the highest GDF-15 tertile. When worsening in the number of Fried frailty criteria and death were combined as an adverse 24-month outcome, the corresponding proportions across the lowest, intermediate, and highest GDF-15 tertiles were 27.8%, 55.4%, and 53.8%, respectively (Fisher’s exact test, *p* = 0.006; **Supplementary Table 2** and **Supplementary Fig 3**).

### 3.2 Longitudinal analyses

**Fig. 1:**
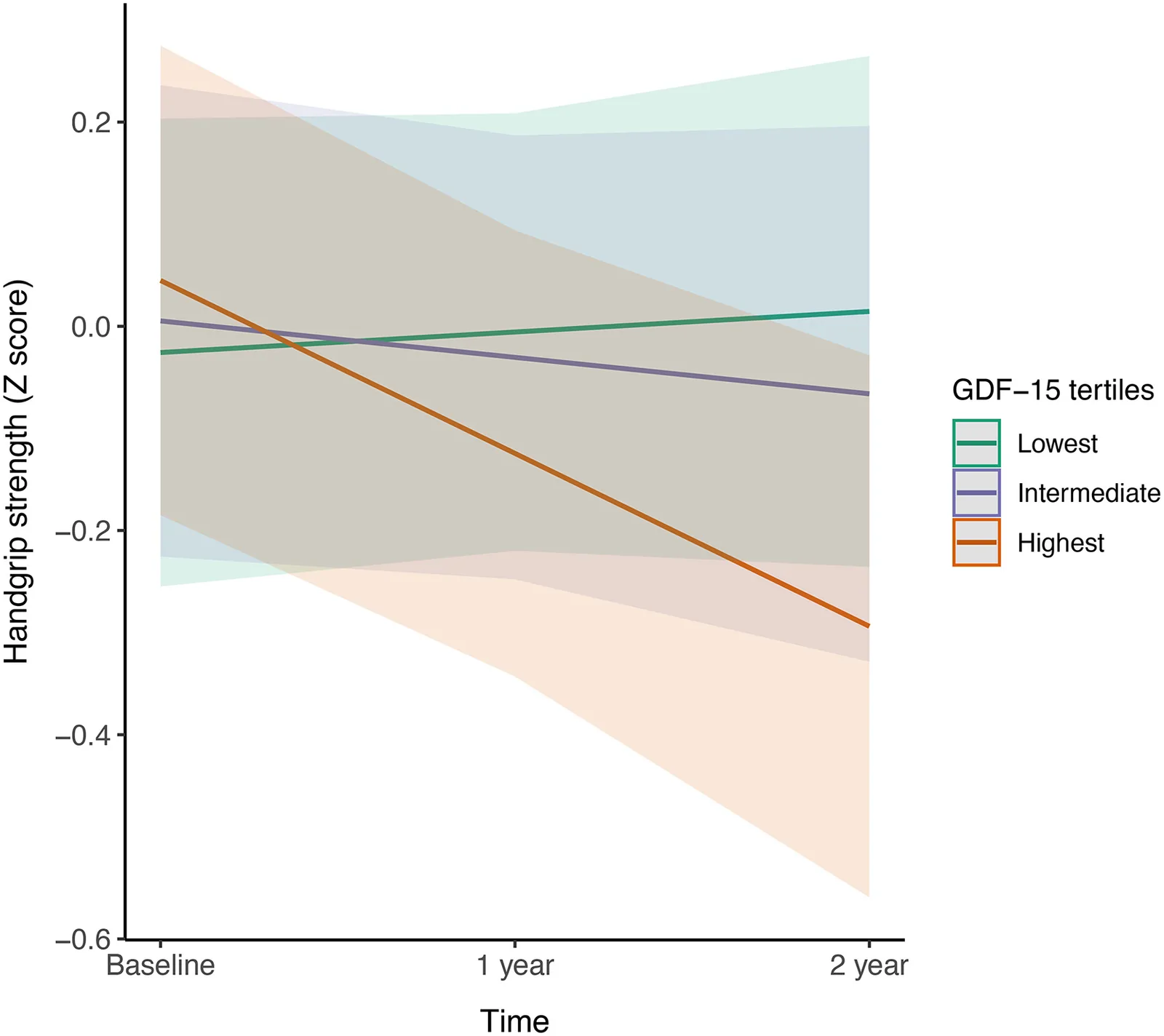
Handgrip strength trajectories over the 2-year follow-up according to GDF-15 tertiles. Legend: GDF-15, Growth Differentiation Factor 15 To account for sex differences in muscle strength, handgrip values were standardized into Z-scores using sex-specific means and standard deviations.

Data about the association between GDF-15 concentrations and longitudinal changes in frailty components are presented in Table 2. Higher GDF-15 levels (continuous) were associated with a steeper decline in handgrip strength over time, whereas no significant associations were observed for number of Frailty items, weight loss, gait speed, sedentary behaviour, or fatigue. When GDF-15 was analysed by tertile, participants in the highest tertile experienced a greater decline in handgrip strength than those in the lowest tertile (Fig. 1). In contrast, no association was observed for the intermediate tertile.

**Table 2:** Associations between GDF-15 concentrations and longitudinal changes in frailty components.

		n of Frailty items			Weight Loss			Muscle strength			Gait speed			Sedentary behaviors				Fatigue		
Plasma GDF-15	n	B	SE	p	SHR	95%CI	p	B	SE	p	B	SE	p	OR	95%CI	p	B		SE	p
GDF-15 as continuous																				
1SD increase	206	0.044	0.057	0.437	1.16	0.92 - 1.47	0.220	-0.071	0.035	**0.047**	-0.019	0.016	0.246	1.09	0.33 - 3.67	0.74	-0.036		0.094	0.702
GDF-15 tertiles																				
Lowest Tertile	69	Ref.			Ref.			Ref.			Ref.			Ref.						
Intermediate Tertile	68	0.326	0.129	**0.013**	2.51	1.04 - 6.04	**0.040**	-0.060	0.080	0.459	0.033	0.035	0.338	1.24	0.58 - 2.65	0.57	0.233		0.217	0.283
Highest Tertile	69	0.206	0.130	0.114	2.19	0.92 - 5.21	0.075	-0.195	0.081	**0.018**	0.002	0.035	0.946	0.79	0.32 - 1.98	0.62	0.049		0.216	0.820

Legend: B, Beta Coefficient, CI, confidence interval; GDF-15, Growth Differentiation Factor 15; OR, Odds Ratio; SD, Standard Deviation; SE, Standard Error; SHR, subdistribution hazard ratio;.

All the models are adjusted by age, sex, living situation (alone vs others), Mini Mental State Examination (MMSE), and Charlson Comorbidity Index (CCI). The model for the number of frailty criteria was additionally adjusted for baseline frailty, whereas the weight loss model was additionally adjusted for baseline BMI.

**Fig. 2:**
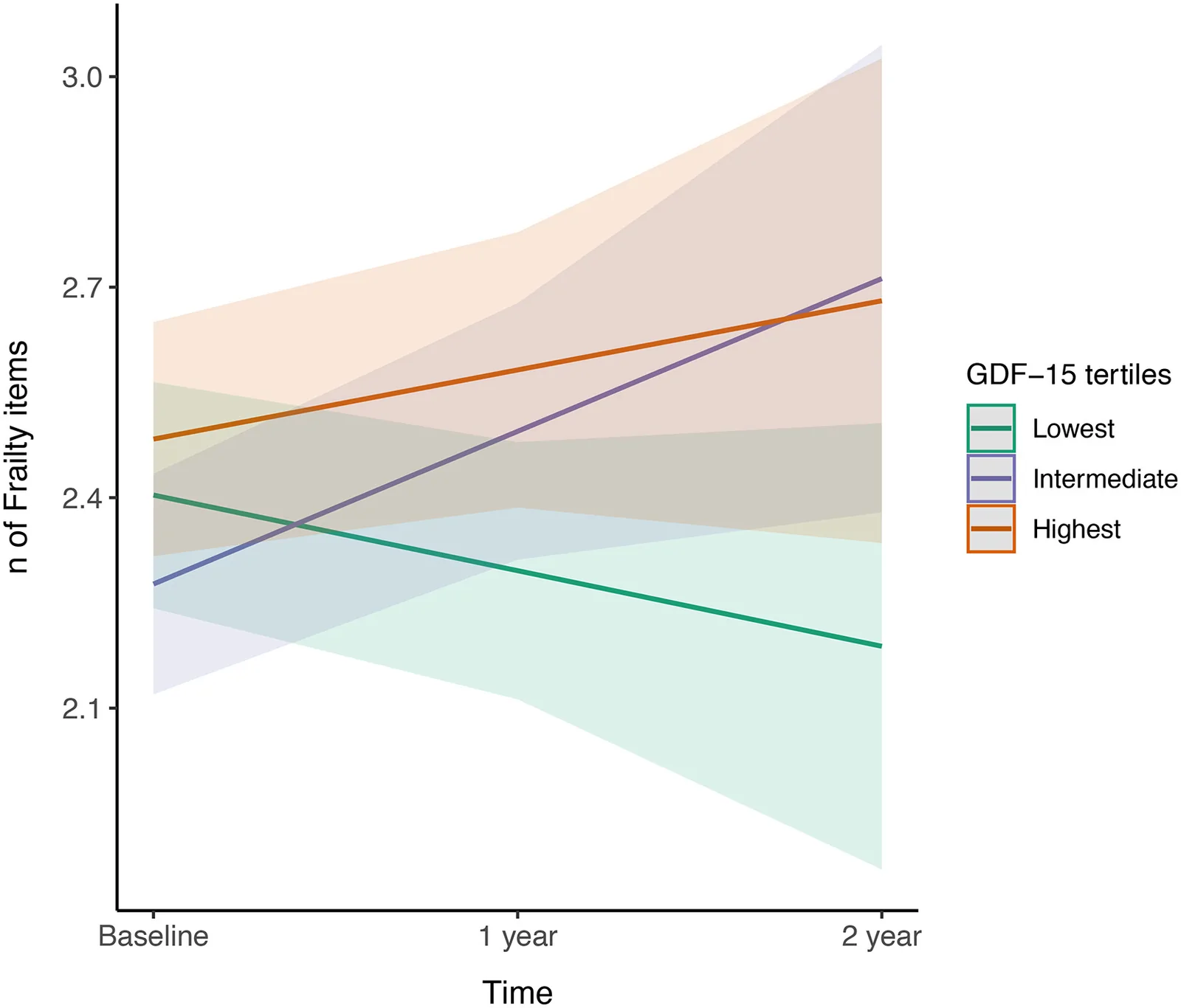
Trajectories of the number of frailty items over the 2-year follow-up according to GDF-15 tertiles. *Legend: GDF-15, Growth Differentiation Factor 15*.

Participants in the intermediate tertile showed a significantly higher risk of worsening frailty, whereas no significant association was observed in the highest tertile. A similar pattern was observed for incident weight loss. At 24 months, the cumulative incidence of weight loss was 11.6%, 25.0%, and 23.2% in the lowest, intermediate, and highest GDF-15 tertiles, respectively. The corresponding cumulative incidence of death before weight loss was 1.4% (n = 1), 7.4% (n = 5), and 13.0% (n = 9), respectively (Fisher’s exact test, *p* = 0.011). Fig. 2

**Fig. 3:**
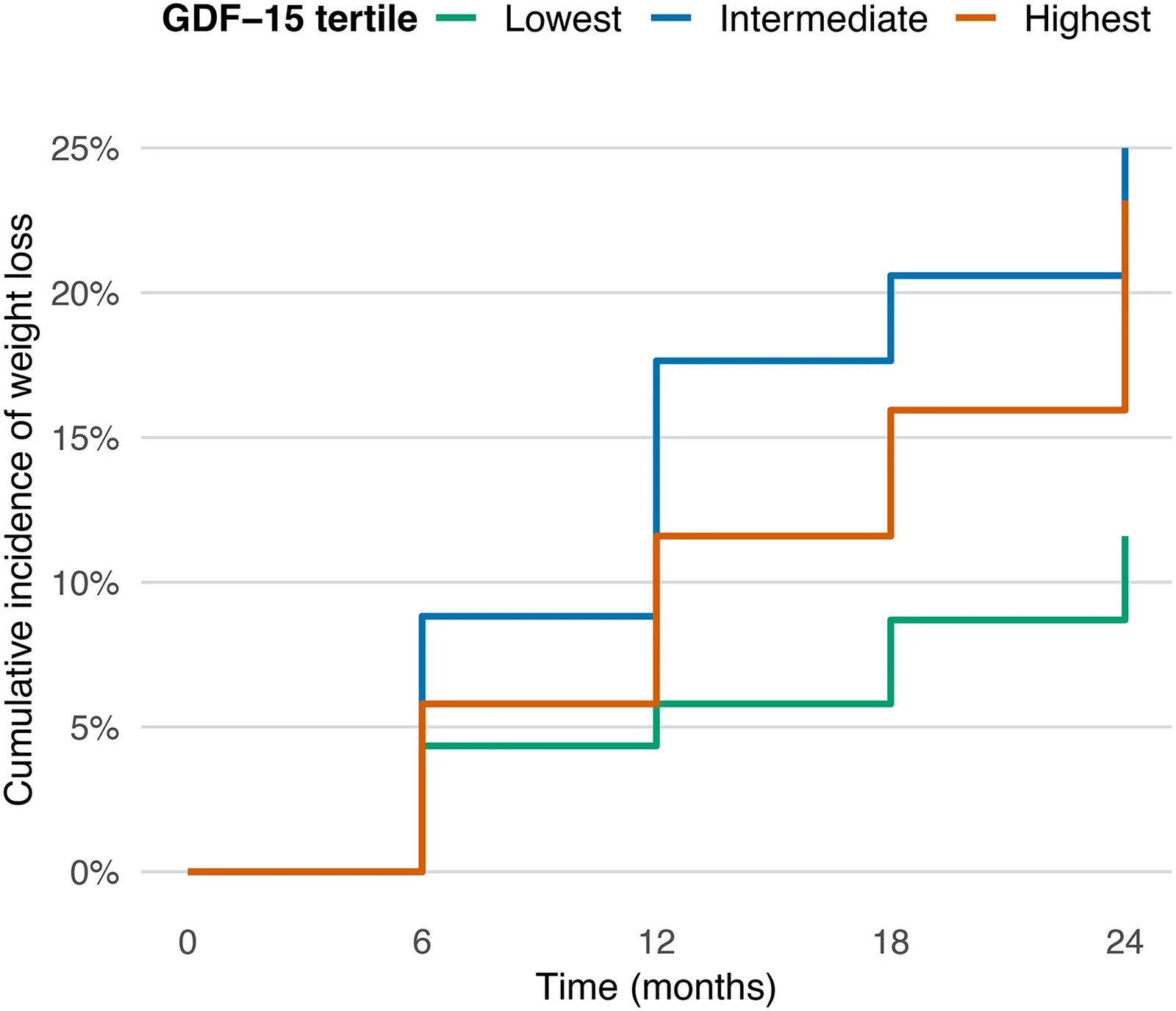
Cumulative incidence of weight loss over the 2-year follow-up according to the GDF-15 tertiles. Weight loss was defined as a ≥ 5% reduction in body weight over six months. *Legend: GDF-15; Growth Differentiation Factor-15; WL, Weight Loss*.

Analyses using exploratory cut-offs are presented in Table 3. Briefly, the 1500 pg/mL threshold confirmed that participants with GDF-15 levels above this value had a significantly higher risk of incident weight loss (Fig. 3). Conversely, the upper tertile threshold (2443 pg/mL) was associated with a steeper decline in muscle strength over time, whereas the 1500 pg/mL cut-off showed only a non-significant trend. No significant associations were found for gait speed, sedentary behaviour, or fatigue.

**Table 3:** Associations between GDF-15 concentrations and longitudinal changes in frailty components using exploratory cut-offs: 1500 pg/mL (eligibility threshold for GDF-15 inhibition in cancer cachexia) and 2443 pg/mL (upper tertile of 6-month distribution).

		n of Frailty items			Weight Loss			Muscle strength			Gait speed			Sedentary behaviors			Fatigue		
Plasma GDF-15	n	B	SE	P	SHR	95%CI	p	B	SE	p	B	SE	p	OR	95%CI	p	B	SE	p
Exploratory threshold 1: 1500 pg/mL																			
Lower GDF-15	60	Ref.			Ref.			Ref.			Ref.			Ref.			Ref.		
Higher GDF-15	146	0.237	0.114	**0.039**	3.35	1.27 - 8.83	**0.015**	-0.131	0.071	0.067	0.048	0.030	0.114	1.08	0.54 - 2.18	0.82	0.159	0.189	0.403
Exploratory threshold 2: upper tertile (2443 pg/mL)																			
Lower GDF-15	137	Ref.			Ref.			Ref.			Ref.			Ref.			Ref.		
Higher GDF-15	69	0.054	0.117	0.641	1.27	0.69 - 2.34	0.450	-0.166	0.071	**0.022**	-0.013	0.031	0.674	0.77	0.36 - 1.65	0.501	-0.058	0.191	0.761

Legend: B, Beta Coefficient, CI, confidence interval; GDF-15, Growth Differentiation Factor 15; OR, Odds Ratio; SD, Standard Deviation; SE, Standard Error; SHR, subdistribution hazard ratio;.

All the models are adjusted by age, sex, living situation (alone vs others), Mini Mental State Examination (MMSE), and Charlson Comorbidity Index (CCI). The model for the number of frailty criteria was additionally adjusted for baseline frailty, whereas the weight loss model was additionally adjusted for baseline BMI.

Sensitivity analyses using restricted cubic splines did not provide statistical evidence of non-linearity for incident weight loss (*p* = 0.699) or the number of Fried frailty criteria (*p* = 0.922).

### 3.3 Secondary analyses

The median change in plasma GDF-15 concentrations between the 6-month and 2-year assessments was +90.0 pg/mL (IQR: −133.0 to +372.0). Longitudinal trajectories of plasma GDF-15 during the follow-up are illustrated in **Supplementary Fig 1 (**Sankey diagram). Regardless of baseline category, about two-thirds of participants remained in the original tertile over the follow-up: 60.0% (n = 30) in the first, 67.5% (n = 27) in the second, and 66.7% (n = 26) in the third. Results of the secondary analyses, conducted on the subgroup with two available GDF-15 measurements (n = 129), are presented in Table 4. Using the upper tertile cut-off (GDF-15 ≥ 2443 pg/mL), participants with persistently above-threshold GDF-15 levels and those with increasing concentrations exhibited a significantly greater decline in handgrip strength compared to those with persistently below-threshold levels (B = - 0.299, SE = 0.088, *p* < 0.001; and B = - 0.493, SE = 0.125, *p* < 0.001, respectively). In contrast, no significant decline was observed among individuals with decreasing concentrations over the follow-up (B = - 0.067, SE = 0.116, *p* = 0.566) (**Supplementary Fig 2**). No associations were observed for other adverse outcomes in this subgroup.

**Table 4:** Associations between GDF-15 concentrations and longitudinal changes in frailty components among the subgroup with two available GDF-15 measurements (n = 129). GDF-15 was treated as a continuous variable (change over time) and categorized into four groups based on changes between the two measurements – “Persistently Low,” “Decreasing,” “Increasing,” and “Persistently High” – using the two exploratory thresholds (1500 pg/mL and 2443 pg/mL).

		n of Frailty items			Weight Loss				Muscle strength				Gait speed				Sedentary behaviors				Fatigue		
Plasma GDF-15	n	B	SE	p	HR	95%CI	p	B		SE	p	B		SE	p	OR		95%CI	p	B		SE	p
GDF-15 as continuous																							
1SD increase	129	0.085	0.115	0.450	1.08	0.70–1.66	0.725	0.051		0.037	0.166	-0.004		0.016	0.795	1.31		0.87–1.98	0.203	0.061		0.097	0.529
Exploratory threshold 1: 1500 pg/mL																							
Persistently Low	27	Ref.			Ref.				Ref.				Ref.				Ref.				Ref.		
Decreasing	5	-0.244	0.325	0.453	1.32	0.15 - 11.61	0.795	0.038		0.020	0.852	-0.004		0.085	0.956	1.15		0.22 - 5.85	0.870	-0.219		0.540	0.687
Increasing	19	-0.024	0.199	0.907	0.42	0.05 - 3.92	0.448	-0.126		0.124	0.311	-0.039		0.052	0.447	1.35		0.44 - 4.19	0.601	-0.229		0.332	0.492
Persistently High	78	0.241	0.149	0.107	2.25	0.69 - 7.36	0.178	-0.218		0.092	**0.019**	0.056		0.039	0.155	1.08		0.46 - 2.53	0.862	0.142		0.248	0.568
Exploratory threshold 2: upper tertile (2443 pg/mL)																							
Persistently Low	79	Ref.			Ref.			Ref.				Ref.				Ref.				Ref.			
Decreasing	13	-0.108	0.201	0.591	1.77	0.67 - 4.71	0.249	-0.067		0.117	0.566	-0.071		0.708	0.920	0.63		0.18 - 2.13	0.456	0.097		0.329	0.769
Increasing	11	0.104	0.217	0.629	1.04	0.37 - 2.91	0.937	-0.494		0.125	**<0.001**	-0.507		0.762	0.507	0.58		0.15 - 2.15	0.415	-0.557		0.355	0.118
Persistently High	26	0.238	0.153	0.121	0.61	0.21 - 1.75	0.359	-0.299		0.088	**<0.001**	-0.295		0.543	0.588	0.69		0.27 - 1.79	0.451	0.212		0.249	0.396

Legend: B, Beta Coefficient, CI, confidence interval; GDF-15, Growth Differentiation Factor 15; OR, Odds Ratio; SD, Standard Deviation; SE, Standard Error, HR, hazard ratio;.

All the models are adjusted by age, sex, living situation (alone vs others), Mini Mental State Examination (MMSE), and Charlson Comorbidity Index (CCI). The model for the number of frailty criteria was additionally adjusted for baseline frailty, whereas the weight loss model was additionally adjusted for baseline BMI.

## 4 Discussion

The main finding of this study is that higher plasma GDF-15 concentrations were longitudinally associated with a steeper decline in handgrip strength among pre-frail and frail older adults with objective cognitive impairment. This association was consistent across different analytical approaches, including continuous models, tertile-based analyses, and exploratory thresholds, and was further supported by analyses using repeated GDF-15 measurements over time. Moreover, individuals in the intermediate tertile showed an increased risk of worsening frailty and incident weight loss, whereas no significant association was observed for the highest tertile. However, these findings should be interpreted with caution, as the highest tertile also showed the greatest competing incidence of death, suggesting that some participants may have died before further frailty worsening or weight loss could be observed. Taken together, these findings suggest that GDF-15 concentrations may be associated with adverse frailty-related trajectories, possibly through declines in muscle strength and incident weight loss, even among individuals who are already physically and cognitively impaired.

Higher plasma GDF-15 concentrations were consistently associated with a steeper decline in handgrip strength, suggesting that this biomarker may identify individuals at heightened risk of progressing toward sarcopenia. Indeed, according to the European Working Group on Sarcopenia in Older People 2 (EWGSOP2) criteria, low muscle strength alone is sufficient to define probable sarcopenia, with impairments in physical performance (e.g., slow gait speed) only indicating more advanced stages of the condition [19]. Previous cross-sectional studies have linked elevated GDF-15 to sarcopenia or muscle weakness [[20], [21], [22], [23]], but longitudinal evidence has been scarce. One study in community-dwelling older adults found that GDF-15 was associated with prevalent sarcopenia but did not predict decline in muscle strength over a two-year follow-up [24]. However, the study used a relatively low GDF-15 cut-off (1245 pg/mL), which may not have been sufficiently elevated to capture the risk of muscle decline - an interpretation further supported by the lack of significant association with the exploratory 1500 pg/mL threshold in our cohort. Biologically, GDF-15 is a marker of mitochondrial stress-response and impaired energy homeostasis [25]. Skeletal muscle, due to its high metabolic demand, is particularly vulnerable to mitochondrial deficits, which can compromise contractile efficiency and accelerate muscle degradation [25]. In addition, elevated GDF-15 has been linked to lower physical activity levels [26], a key driver of muscle wasting [27]. Even in our cohort, participants in the highest GDF-15 tertile showed a trend toward greater sedentary behaviour at baseline, suggesting that lower activity levels may have contributed, at least in part, to the observed decline in muscle strength among individuals with elevated GDF-15.

In contrast, individuals in the intermediate tertile showed an increased risk of incident weight loss, whereas only a trend was observed for the highest tertile. While the association may be partially explained by the action of GDF-15 on appetite-regulating circuits in the central nervous system, leading to appetite loss and reduced food intake [28], the weaker association at higher concentrations was unexpected. One possible explanation could have been attributable to the intrinsic characteristics of the weight loss process, which tends to plateau after a certain degree of reduction. However, our sensitivity analyses using cubic splines showed no statistical evidence of non-linearity, making this interpretation less likely. At the same time, the highest GDF-15 tertile showed a high cumulative incidence of weight loss (almost one fourth of the group) and the highest competing incidence of death. Therefore, the lack of conventional statistical significance in the highest tertile should be interpreted with caution. Very high GDF-15 levels reflect greater overall vulnerability, and it is plausible that some frail participants died before weight loss could be observed. In addition, the relatively small sample size, which was further reduced by the higher mortality in this group, may have limited the ability to detect a statistically significant association. Finally, participants in the COGFRAIL cohort were receiving geriatric care, and those considered at higher risk of weight loss - or who had already lost weight - may have been offered nutritional supplementation or physical interventions, which could have influenced their subsequent weight loss trajectory.

A similar interpretation may apply to frailty worsening over time. Participants in the intermediate - but not those in the highest tertile - had a significantly greater risk of worsening frailty during follow-up. In light of the previous findings, this association may partly reflect the contribution of incident weight loss and decline in muscle strength, both of which are central components of the frailty phenotype. However, as observed for weight loss, the absence of a significant association in the highest tertile was unexpected. Descriptive analyses showed that more than half of participants in both the intermediate and highest tertiles experienced either frailty worsening or death by 24 months, with the greatest number of deaths occurring in the highest tertile. This pattern suggests that the absence of statistical significance in this tertile should not be interpreted as absence of vulnerability. Rather, very high GDF-15 concentrations may identify individuals with more advanced biological vulnerability, in whom death may occur before further worsening of the frailty phenotype can be observed. This interpretation, although speculative, is consistent with the deficit accumulation model of frailty, which suggests that there may be a practical upper limit to the degree of frailty that can be accumulated before death occurs [29].

The absence of longitudinal association between GDF-15 and gait speed contrasts with previous findings from a 9-year study in older adults showing that higher GDF-15 predicted incident mobility disability, defined as the inability to complete a 400-meter walking test [30]. It is plausible that the two-year follow-up period in our study was sufficient to capture early declines in muscle strength but too short to detect significant declines in physical performance. In this context, muscle strength decline may represent an earlier manifestation of frailty progression, whereas changes in gait speed or mobility disability may require longer follow-up to become clinically detectable. Similarly, the lack of associations with sedentary behaviour and fatigue should be interpreted cautiously. A high proportion of participants already exhibited sedentary behaviour (74.3%) and fatigue (81.9%) at baseline, resulting in limited variability over time. Moreover, these measures were self-reported, and the sedentary behaviour scale required dichotomisation due to skewed distribution, which may have reduced sensitivity to change. Future studies using objective and more granular measures of physical activity and sedentary behaviour, such as accelerometry, may help clarify whether GDF-15 is associated with changes in these domains.

In the secondary analyses, we observed that approximately one-third of participants changed GDF-15 tertile over the two-year follow-up, regardless of their baseline category, suggesting that repeated measurement of GDF-15 concentrations may better capture individual variability over time. Furthermore, using an exploratory cut-off based on the tertile distribution of our sample, we found that not only participants with persistently high GDF-15 levels, but also those whose levels increased during follow-up, exhibited a steeper decline in handgrip strength. In contrast, this association was not observed among individuals whose GDF-15 levels decreased over time. These findings may suggest that interventions aimed at reducing GDF-15 concentrations could potentially prevent muscle strength decline. However, given the small sample size in these secondary analyses and the exploratory nature of the cut-offs, these results should be interpreted with caution.

From a clinical perspective, our results suggest that GDF-15 could help identify individuals at risk of weight loss or muscle strength decline, even within pre-frail or frail populations. Mild elevations of GDF-15 might indicate the need for nutritional assessment and weight monitoring, whereas higher concentrations may signal broader clinical vulnerability, including higher mortality and greater susceptibility to muscle deterioration, supporting early implementation of resistance training or protein supplementation [31]. The potential integration of GDF-15 into prognostic models could help tailor interventions to individual biological risk profiles. Although the exploratory 1500 pg/mL cut-off was originally identified in cancer cachexia as an eligibility threshold for GDF-15 inhibitor treatment, even in our cohort of pre-frail and frail older adults, participants above this value had a higher risk of developing weight loss. However, the high proportion of participants above this threshold (71%) suggests that the same cut-off may not carry the same biological or clinical meaning in geriatric populations as it does in cancer cachexia. Therefore, further validation is needed before this threshold can be used to identify older adults who may benefit from GDF-15–targeted interventions.

Our study has several strengths. First, because patients were recruited from outpatient visits to Frailty or Memory Clinics as part of their routine assessments, the sample is highly representative of real-world populations. Moreover, the longitudinal follow-up enabled the definition of a temporal relationship between GDF-15 elevation and adverse outcomes, and the repeated assessment of GDF-15 levels permitted the differentiation between punctual and persistent elevation of this biomarker. Nonetheless, some limitations should be acknowledged. First, this is a secondary analysis of the COGFRAIL study, which was not originally designed to address this research question. Second, GDF-15 was first measured at the 6-month visit rather than at the initial COGFRAIL baseline. Therefore, baseline clinical characteristics refer to the first visit, whereas GDF-15 concentrations refer to the 6-month assessment, creating a temporal mismatch that may have introduced potential misclassification. Third, to avoid overfitting, models were not adjusted separately for individual conditions that may be associated with plasma GDF-15 concentrations, such as chronic kidney disease or heart failure [32]. Instead, we adjusted for the CCI, which provides a composite measure of comorbidity burden and includes these conditions. However, the renal component of the CCI captures only moderate-to-severe renal impairment and may not fully account for the continuous, graded association between GDF-15 concentrations and renal function [33]. Therefore, residual confounding by kidney function cannot be excluded. In addition, although the analyses were interpreted according to a hierarchical framework, the number of statistical comparisons may have increased the risk of false-positive findings and should therefore be considered when interpreting the results. Finally, the sample size was modest, particularly for secondary analyses, and a substantial proportion of participants did not complete the 2-year follow-up. Among the 206 participants included in the analytical sample, 77 did not complete follow-up because of death, withdrawal of consent, medical problems, institutionalisation, or other reasons. This may have introduced selection bias and reduced the ability to detect longitudinal associations, especially among the most vulnerable participants.

In conclusion, in this cohort of pre-frail and frail older adults, higher plasma GDF-15 concentrations consistently predicted a steeper decline in muscle strength over time. Approximately one-third of participants changed GDF-15 category over two years, and individuals with persistently elevated or increasing GDF-15 levels experienced the greatest decline in muscle strength. GDF-15 also showed prognostic value for incident weight loss and worsening frailty, although these associations appeared to attenuate at higher concentrations, possibly because participants with very high GDF-15 levels were more likely to die before these outcomes could be observed. Future studies should further investigate the mechanisms linking GDF-15 to physical decline and appetite in older adults, to determine whether modulating this pathway could help prevent or slow the deterioration associated with frailty.

## Ethical guidelines statement

The present work follow the Ethical guidelines for authorship and publishing in the Journal of Cachexia, Sarcopenia and Muscle

## Declaration of generative AI and AI-assisted technologies in the writing process

The authors used ChatGPT (OpenAI) to assist with minor language editing and correction of English grammar. The tool was not used for conceptual development, data analysis, or interpretation of the results.

## Funding

The COGFRAIL study has obtained funding from MSDAVENIR. This work was performed into the context of the IHU HealthAge, which has benefited from funding by the Agence Nationale de la Recherche under the France 2030 program (reference number: ANR-23-IAHU-0011).

## Data sharing

De-identified data from the COGFRAIL cohort are available to researchers upon request, following approval of a methodologically sound research proposal and signature of a data use agreement. Enquiries or proposals should be addressed to sourdet.s@chu-toulouse.fr.

## CRediT authorship contribution statement

**Federico Bellelli:** Writing – original draft, Formal analysis. **Juan Luis Sanchez Sanchez:** Writing – review & editing, Supervision, Methodology. **Davide Angioni:** Writing – review & editing, Supervision, Conceptualization. **Alexandre Lucas:** Writing – review & editing, Data curation. **Yves Rolland:** Writing – review & editing, Conceptualization. **Marco Proietti:** Writing – review & editing, Supervision, Conceptualization. **Bruno Vellas:** Conceptualization, Writing – review & editing. **Philipe De Souto Barreto:** Writing – review & editing, Supervision, Methodology, Conceptualization. **Sandrine Sourdet:** Writing – review & editing, Supervision, Data curation.

## Declaration of competing interest

The authors declare that they have no known competing financial interests or personal relationships that could have appeared to influence the work reported in this paper.

## Appendix Supplementary materials

Image, application 1
