# Supplementary material for: Association of plasma growth differentiation factor-15 with the frailty phenotype – Evidence from the COGFRAIL study
Source: J Frailty Aging. 2026 Sep 4;15(5):100194. doi: 10.1016/j.tjfa.2026.100194 (PMC13572050; doi:10.1016/j.tjfa.2026.100194)
Supplement: Supplementary file 1 [file mmc1.docx]

**Supplementary Table 1. Comparison of Fine–Gray and Cox regression models for the association between GDF-15 concentrations and incident weight loss**

|  |  | Weight Loss | | | Weight Loss | | |
| --- | --- | --- | --- | --- | --- | --- | --- |
| Plasma GDF-15 | n | SHR | 95%CI | p | HR | 95%CI | p |
|  | | | | | | | |
| 1SD increase | 206 | 1.16 | 0.92 - 1.47 | 0.220 | 1.07 | 0.85 - 1.34 | 0.559 |
|  | | | | | | | |
| Lowest Tertile | 69 | Ref. | | | Ref. | | |
| Intermediate Tertile | 68 | 2.51 | 1.04 - 6.04 | **0.040** | 2.56 | 1.07 - 6.16 | **0.035** |
| Highest Tertile | 69 | 2.19 | 0.92 - 5.21 | 0.075 | 2.01 | 0.79 - 5.12 | 0.141 |

*Legend: CI, confidence interval; GDF-15, Growth Differentiation Factor 15; HR, hazard ratio; SHR, subdistribution hazard ratio;*

*All the models are adjusted by age, sex, living situation (alone vs others), Mini Mental State Examination (MMSE), Charlson Comorbidity Index (CCI), and baseline BMI.*

**Supplementary Table 2. Changes in Fried frailty criteria and death at 24 months according to baseline GDF-15 tertiles.**

|  | Lowest GDF-15 tertile | Intermediate GDF-15 tertile | Highest GDF-15 tertile |
| --- | --- | --- | --- |
| Improved | 24 (44.4%) | 11 (23.4%) | 11 (21.2%) |
| Stable | 15 (27.8%) | 10 (21.3%) | 13 (25.0%) |
| Worsened | 14 (25.9%) | 20 (42.6%) | 18 (34.6%) |
| Dead before 24 months | 1 (1.9%) | 6 (12.8%) | 10 (19.2%) |
| Worsened or dead | 15 (27.8%) | 26 (55.4%) | 28 (53.8%) |

**Legend:** Values are n (%). Changes in Fried frailty criteria were classified between baseline and 24 months. Death before the 24-month assessment was reported as a separate category. “Worsened or dead” was calculated as a composite adverse 24-month outcome.

**Supplementary Figure 1:** Sankey diagram illustrating transitions between GDF-15 tertiles over time

*Legend: GDF-15, Growth Differentiation Factor 15*

**Supplementary Figure 2:** Handgrip strength trajectories over the 2-year follow-up according to the upper tertile cut-off (2443 pg/mL) for people with both baseline and 2-year GDF-15 measurement (n=129).

*Legend: GDF-15, Growth Differentiation Factor 15*

**Supplementary Figure 3.** Distribution of changes in Fried frailty criteria and death at 24 months according to baseline GDF-15 tertiles.
